# Management of Severe COVID-19 in Pregnancy

**DOI:** 10.1155/2020/8852816

**Published:** 2020-07-27

**Authors:** Yassamine Abourida, Houssam Rebahi, Imane Oussayeh, Hajar Chichou, Bouchra Fakhir, Abderraouf Soummani, Hicham Jalal, Fatiha Bennaoui, Nadia El Idrissi Slitine, Fadl Mrabih Rabou Maoulainine, Ahmed Rhassane El Adib, Mohamed Abdenacer Samkaoui

**Affiliations:** ^1^Department of Anesthesia & Intensive Care Medicine, Faculty of Medicine and Pharmacy of Marrakech, Cadi Ayyad University, Marrakech, Morocco; ^2^Department of Obstetrics & Gynecology, Mother & Child Hospital, Mohammed VI University Hospital of Marrakech, Morocco; ^3^Department of Radiology, Mother & Child Hospital, Mohammed VI University Hospital of Marrakech, Morocco; ^4^Department of Neonatal Intensive Care, Mother & Child Hospital, Mohammed VI University Hospital of Marrakech, Morocco

## Abstract

The scarcity of data concerning pregnant patients gravely infected with severe acute respiratory syndrome coronavirus 2 (SARS-CoV-2) makes their management difficult, as most of the reported cases in the literature present mild pneumonia symptoms. The core problem is laying out evidence on coronavirus's implications on pregnancy and delivery, as well as vertical transmission and neonatal mortality. A healthy 30-year-old pregnant woman, gravida 6, para 4, at 31 weeks of gestation, presented severe pneumonia symptoms promptly complicated with premature rupture of membranes (PROM). A nasopharyngeal swab returned positive for SARS-CoV-2 using reverse transcription polymerase chain reactions (RT-PCR). The parturient underwent a cesarean delivery. This paper is an attempt to outline management of the critical condition of COVID-19 during pregnancy.

## 1. Introduction

In the early months of 2020, the world witnessed a rapid spread of the severe acute respiratory syndrome coronavirus 2 (SARS-Cov-2). COVID-19 became the most threatening global health crisis of our time. During this outbreak period, pregnant women and their newborns were also afflicted significantly. Limited data describe the majority of parturients as having mild symptoms of COVID-19 such as fever, cough, and shortness of breath but report only a few severe cases [1–3].

It has not yet been established whether coronavirus can induce preterm birth or intrauterine infection. As the event of pregnancy represents a compromised state of immunity, the implication of COVID-19 on the pathophysiology is yet to be understood, along with epidemiological features and prognosis in late-term pregnancy. It is relevant to mention that no concrete evidence determined the risk of vertical transmission.

Herein, we outline a case of severe COVID-19 infection in a pregnant woman abruptly rupturing her membranes and undergoing cesarean delivery.

## 2. Case Report

A healthy 30-year-old pregnant woman, gravida 6, para 4 (1 fetal death), with a history of premature rupture of membranes (PROM) at 20 weeks in a previous gestation, a scarred uterus, and osteoarthritis, was admitted, at 31 weeks of gestation, to the obstetric emergency room 4 days after the onset of the following symptoms: shortness of breath, fever at 38.5°C, and persistent dry cough promptly complicated with PROM.

**Figure 1:**
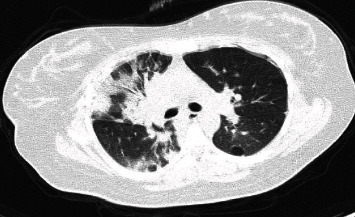
Chest computed tomography scan of the mother.

Physical examination revealed tachypnea with a respiratory rate (RR) of 30 breaths/min, a 92% oxygen saturation (SpO_2_), tachycardia with a heart rate (HR) of 109 beats/min, and blood pressure of 96/50 mmHg. She was quickly put on nasal cannula oxygen support at a flow of 3 L/min and improved her oxygen saturation to 98%. Her uterine height was 28 cm with no sign of uterine contraction. Vaginal examination demonstrated ruptured membranes and a cephalic presentation. Ultrasound was performed, showing active fetal movements, normal fetal morphology, normal amniotic fluid quantity, and an estimated fetal weight of 1960 g. A chest computed tomography scan detected bilateral condensation opacities reaching 50 to 75 percent suggesting a viral infection, as shown in Figure 1. A nasopharyngeal swab returned positive for SARS-CoV-2 using reverse transcription polymerase chain reactions (RT-PCR).

Healthcare professionals wore appropriate personal protective equipment (PPE), according to each level of contamination risk. The patient was hospitalized in an isolated room in the reserved COVID-19 maternity ward, wearing a surgical mask during her stay. Initially, the patient received intravenous fluids, acetaminophen, and prophylactic 3^rd^-generation cephalosporin (ceftriaxone). The patient also received corticosteroids (betamethasone) to ensure fetal pulmonary maturation. A complete blood panel demonstrated an intense inflammatory status displayed by hyperferritinemia and an elevated level of CRP as shown in Table 1.

**Figure 2:**
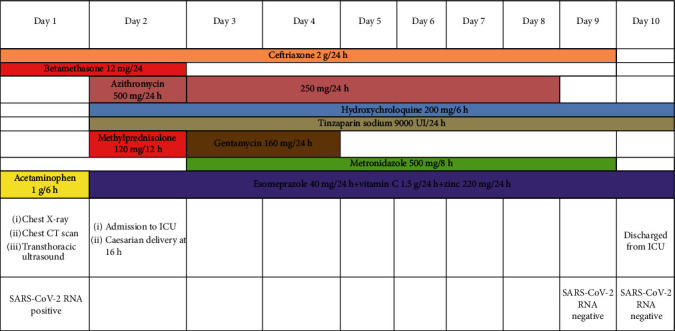
Therapeutic arsenal received by the mother.

**Figure 3:**
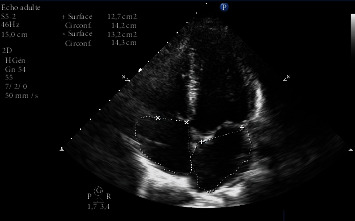
Transthoracic echocardiography of the mother.

After 24 hours, the patient became hypoxemic with a SpO_2_ of 89%, RR of 38/min, and hyperthermia of 39.0°C. She was instantly admitted to the COVID-19 intensive care unit (ICU). She was administered high-flow oxygen (10 L/min) via a non-rebreather mask and placed in a lateral decubitus position. She received azithromycin, hydroxychloroquine, and a curative dose of tinzaparin sodium (Figure 2), and as she failed to improve, we added methylprednisolone 1 mg/kg/day. Echocardiogram did not find any abnormalities (Figure 3).

**Figure 4:**
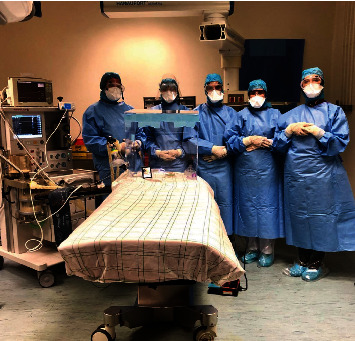
Healthcare workers wearing personal protective equipment in the operating theater, University Hospital of Mohammed VI, Marrakech.

The patient reported intense and frequent uterine contractions as she went into labor. The fetal heart rate monitoring found acute fetal distress. Consequently, an urgent caesarian section was performed. The healthcare team transported the patient, following the COVID-19-dedicated hallways, to an operating room with negative pressure.  Figure 4 portrays anesthesiologists and an obstetrician surgeon wearing level 3 PPE and an aerosol protective shield for a potential intubation. The patient received oxygen support on nasal cannula at flow of 5 L/min while being routinely monitored. She underwent single-shot spinal anesthesia using bupivacaine (10 mg), morphine (100 *μ*g), and fentanyl (25 *μ*g). A lower segment cesarean section was performed without any incident, as she remained stable.

Postoperative pain was handled by spinal morphine analgesia that lasted 16 hours. The patient improved her respiratory function with an RR of 15 breaths/min, a SpO_2_ of 94%, and a temperature of 38.2°C. However, vaginal examination found foul-smelling mucopurulent lochia associated with a delayed uterine involution and an increased serum level of CRP to 204.2 mg/L.

Under the light of these findings, we suspected postpartum endometritis which was treated by an association of metronidazole and gentamycin, along with prior ceftriaxone. After 7 days, the patient's evolution was marked by a full clinical recovery; her last blood panel showed a decreased serum level of CRP to 37.42 mg/L with a white blood cell count of 10.16 × 10^9^/L. She was discharged to the maternity ward, after being negative for SARS-CoV-2 nasopharyngeal swab two days in a row.

The premature newborn, weighted 1700 g with an Apgar score of <7 at 1 min and at 5 min. The neonate was immediately isolated and transported to the COVID-19 neonatal intensive care unit; no skin-to-skin contact was made with the mother. The newborn experienced severe asphyxia, failing to improve under ventilation. At 1 hour of life, a nasopharyngeal swab viral test was performed, returning negative for SARS-CoV-2 using RT-PCR. The initial blood panel, as portrayed Table 2, demonstrated a white blood cell count of 21.27 × 10^9^/L with a CRP of 90.29 mg/L, whereas blood culture remained sterile. The neonate's condition continued to deteriorate due to septic shock and respiratory failure and died after 5 days of birth.

## 3. Discussion

Herein, we report the case of a healthy parturient infected with SARS-CoV-2 in her third trimester, whose condition deteriorated leading to premature rupture of membranes, a premature birth via a caesarian delivery, and neonatal death. Limited data is available on the effect of COVID-19 among pregnant patients and neonates.

In a series of 119 cases, only 6 patients required ICU admission, the rest presented no or mild pneumonia symptoms [1]. Two cases described COVID-19-related cardiomyopathy [2]. The correlation between preterm birth and COVID-19 remains unsettled. A recent meta-analysis suggested that COVID-19 infection was associated with a relatively higher rate of preterm birth, cesarean delivery, and perinatal death [3].

It is unclear if SARS-Cov-2 causes placenta-induced disturbances; however, predilection for thrombosis in the fetal circulation was related to maternal COVID-19 infection as a preliminary finding [4]. Clearly there may be other possible explanations; the exacerbated COVID-19 response, due to proinflammatory cytokines, alters the fragile balance between a controlled immune response and a host-damaging reaction [5]. Furthermore, Cytokine Releasing Syndrome (CRS) might play a pivotal role in a patient condition's deterioration. Ferritin is judged as a proinflammatory signaling molecule that may compromise the immune system during pregnancy. Ferritin could exert a pathogenic role in CRS-related conditions, rather than being just the result of hyperinflammation [6, 7]. Perhaps ferritin could be used as a biological marker for prediction of clinical deterioration during third-term pregnancies. Further evidence would be needed to determine exactly how proinflammatory signals reflect the well-being of a pregnancy.

With the data shortage concerning management of COVID-19 critically ill late pregnancies, each case should be handled apart and clinical decisions should be discussed in a multidisciplinary approach including anesthesiologists, maternal-fetal doctors, neonatologists, and pulmonary intensive care doctors. There is currently no evidence to favor one mode of birth over another; scholarly societies [3, 8] have concluded that COVID-19 is not a contraindication to vaginal delivery. However, the main limitation is that during labor, pregnant women may generate a large number of droplets and aerosols [9]. Knowing that COVID-19 is highly infectious, with an estimated basic reproduction number (R0 = 3.36) [10], a vaginal delivery may increase the risk of exposure and infection of medical staff. Ultimately, it could also present an infection route for SARS-CoV-2 through feces [11], especially for the newborn. But nonetheless, respiratory function should essentially be taken into consideration, as well as obstetric indications as usual. COVID-19 infection is not an argument on its own for a caesarian delivery. Bearing in mind that due to the pathophysiological changes during pregnancy, parturient with pneumonia can easily progress to severe disease, increasing maternal complications [12]. The decision of caesarian delivery of our patient was made considering many factors in favor: hyperinflammation status, PROM, scarred uterus, and fetal distress, resulting presumably from severe hypoxemic pneumonia, cytokine storm, and amniotic fluid infection.

Hydroxychloroquine (HCQ) associated with azithromycin was prescribed, resulting in no adverse effect, following the national therapeutic guidelines [13]. HCQ was found to be safe to use during pregnancy [14]. Initial studies have demonstrated good virological and clinical outcomes with HCQ therapy, with or without azithromycin [15, 16].

To the best of our knowledge, no evidence proved that epidural or spinal analgesia or anesthesia is contraindicated in the presence of coronaviruses. Epidural analgesia should therefore be recommended in labor, to women with suspected or confirmed COVID-19 [8]. Nevertheless, it was noted that 86% of parturients with COVID-19 undergoing epidural anesthesia experienced intraoperative hypotension [17]. It seems plausible to correlate the intraoperative hypotension with the binding of SARS-CoV-2 spike glycoprotein with ACE2 (angiotensin-converting enzyme) receptors that are mainly expressed in blood vessels and lungs [10]. General anesthesia did not have any adverse effects on the neonatal outcomes and mothers' recovery from COVID-19 [17]. During endotracheal intubation, strict precautions should be taken by anesthesiologists, such as the use of an intubation shield box as a protective means against generated aerosol and the application of an antiviral filter filters between the face mask and the manual ventilation device. Medical staff safety must be ensured; therefore, a dedicated COVID-19 circuit must be established and used with every suspected parturient, and this includes triage unit, maternity ward, hallways, operating theater, and expectance and delivery room.

There is a challenging problem concerning vertical transmission of SARS-CoV-2, as conflicting evidence is growing [18–21]. Prior studies have failed to demonstrate maternal-fetal transmission of SARS-CoV-2, including negative testing in amniotic fluid, umbilical cord blood, vaginal swabs, and breast milk [22–24]. Furthermore, some studies have shown that there is no evidence of intrauterine infection caused by vertical transmission in women who develop COVID-19 in late pregnancies [23], while other observations indicate that coronaviruses may cause early pregnancy loss [25]. It is noteworthy that recent reports highlighted elevated SARS-CoV-2 antibody levels (IgM and IgG) and abnormal cytokine test results 2 hours after birth in a neonate born to a mother with COVID-19 via a caesarian delivery, whereas RT-PCR tests on nasopharyngeal swabs taken were negative [20].

Extreme caution must be taken when associating intrauterine infection and serological findings, as well as serious attention must be paid to strict isolation methods during labor to avoid perinatal hospital infection transmission. Further work needs to be carried out to enlighten the repercussions of COVID-19 during pregnancy, as well as its management in the ICU.

Limitations of this report are that it is based on a single case, the neonate antibodies were not analyzed, and the autopsy was not performed. Unfortunately, we were unable to investigate the novel coronavirus pathology hallmark in the placenta.

## 4. Conclusion

Management of parturients infected with SARS-CoV-2 should be planned ahead, and professionals must be trained to take extreme precautions assisting patients in delivery. During the coronavirus pandemic, it became vital to prevent intrahospital exposure in the high-risk population such as pregnant women and newborns. This case may imply that vertical transmission is unlikely, but it cannot be ruled out as the pathogenesis and the impact of SARS-CoV-2 on the placenta are imprecise. Further evidence is still necessary to provide substantial answers.

## Figures and Tables

**Table 1:** Laboratory results of the mother.

	Day 1 27/04	Day 2 28/04	Day 3 29/04	Day 5 01/05	Day 7 03/05	Day 8 04/05	Reference range
White blood cell count (×10^9^/L)	12.82	12.73	18.85	12.75	10.16	10.16	4-10
Ly T CD3 (×10/mm^3^)	108.4	—	—	153.3	—	176.2	100-220
Ly T CD4 (×10/mm^3^)	64.7	—	—	96.3	—	102.5	53-130
Ly T CD8 (×10/mm^3^)	37.8	—	—	50.4	—	64.5	33-92
Ly B (×10/mm^3^)	37.8	—	—	51.8	—	64.5	11-57
Ly NK (×10/mm^3^)	11.4	—	—	14.7	—	20.9	7-48
C-reactive protein (mg/L)	288.9	204.2	131.87	214.09	87.36	37.42	0-5
Ferritine (ng/mL)	422	585	556	417	253	—	30-400
Lactate dehydrogenase (U/L)	237	294	329	301	260	—	0-250
D-dimer (*μ*g/mL)	3.64		2.53	1.89		—	0-0.50
Troponin (pg/L)		4.02	10.25	4.89	3.54	—	0-13
PCT (ng/mL)		0.42			0.22	—	0.1

Ly: lymphocyte; PCT: procalcitonin.

**Table 2:** Laboratory results of the neonate.

	Value	Reference range
White blood cell count (×10^9^/L)	21.27	4-10
Neutrophil count (×10^9^/L)	14.09	2-7.5
Neutrophil ratio (%)	66.2	—
Lymphocyte count (×10^9^/)	3.02	1-4
Lymphocyte ratio (%)	14.2	—
C-reactive protein (mg/L)	90.29	0-5
Total bilirubin (mg/L)	92.3	0-10
Direct bilirubin (mg/L)	4.1	0-3
Creatine kinase (mg/L)	18.6	7-12
Blood culture	Sterile	—
